# Supplementary material for: A single cell atlas of the human liver tumor microenvironment
Source: Mol Syst Biol. 2020 Dec 17;16(12):e9682. doi: 10.15252/msb.20209682 (PMC7746227; doi:10.15252/msb.20209682)
Supplement: Supplementary file 1 — Appendix [file MSB-16-e9682-s001.docx]

**Appendix for:**

**A single cell atlas of the human liver tumor microenvironment**

Hassan Massalha^1^, Keren Bahar Halpern^1^, Samir Abu-Gazala^2,3^, Tamar Jana^1^, Efi E. Massasa^1^, Andreas E. Moor^4^, Lisa Buchauer^1^, Milena Rozenberg^1^, Eli Pikarsky^5^, Ido Amit^6^, Gideon Zamir^2^, Shalev Itzkovitz^1*^

^1^Department of Molecular Cell Biology, Weizmann Institute of Science, Rehovot, Israel.

^2^Department of General Surgery, Hadassah Hebrew University Medical Center, Jerusalem, Israel.

^3^Transplant Division, Department of Surgery, Hospital of the University of Pennsylvania, Philadelphia, PA, USA.

^4^Department of Biosystems Science and Engineering, ETH Zurich, Basel, Switzerland.

^5^The Lautenberg Center for Immunology, Institute for Medical Research Israel-Canada, Hebrew University Medical

School, Jerusalem, Israel.

^6^Department of Immunology, Weizmann Institute of Science, Rehovot, Israel.

*To whom correspondence should be addressed. E-mail: [shalev.itzkovitz@weizmann.ac.il](mailto:shalev.itzkovitz@weizmann.ac.il)

Table of content

[Appendix Figures 2](#_Toc55819067)

[Appendix Figure S1 – Patient distribution across all clusters 2](#_Toc55819068)

[Appendix Figure S2 – smFISH validations of the expression of key cell-type specific markers 3](#_Toc55819069)

[Appendix Figure S3 – Stability analysis of the cluster gene expression signatures. 4](#_Toc55819070)

[Appendix Figure S4 – Immune cell type annotation 5](#_Toc55819071)

[Appendix Figure S5 – Markers of the mesenchymal cell types 6](#_Toc55819072)

[Appendix Figure S6 – Matrisome analysis 8](#_Toc55819073)

[Appendix Figure S7 – Hepatocytes spatial reconstruction 9](#_Toc55819074)

[Appendix Figure S8 – Spatial reconstruction validation, transcription factors and surface markers 10](#_Toc55819075)

[Appendix Figure S9 – Comparison of Kegg pathways enrichment analysis between human and mouse 11](#_Toc55819076)

[References 12](#_Toc55819077)

# Appendix Figures


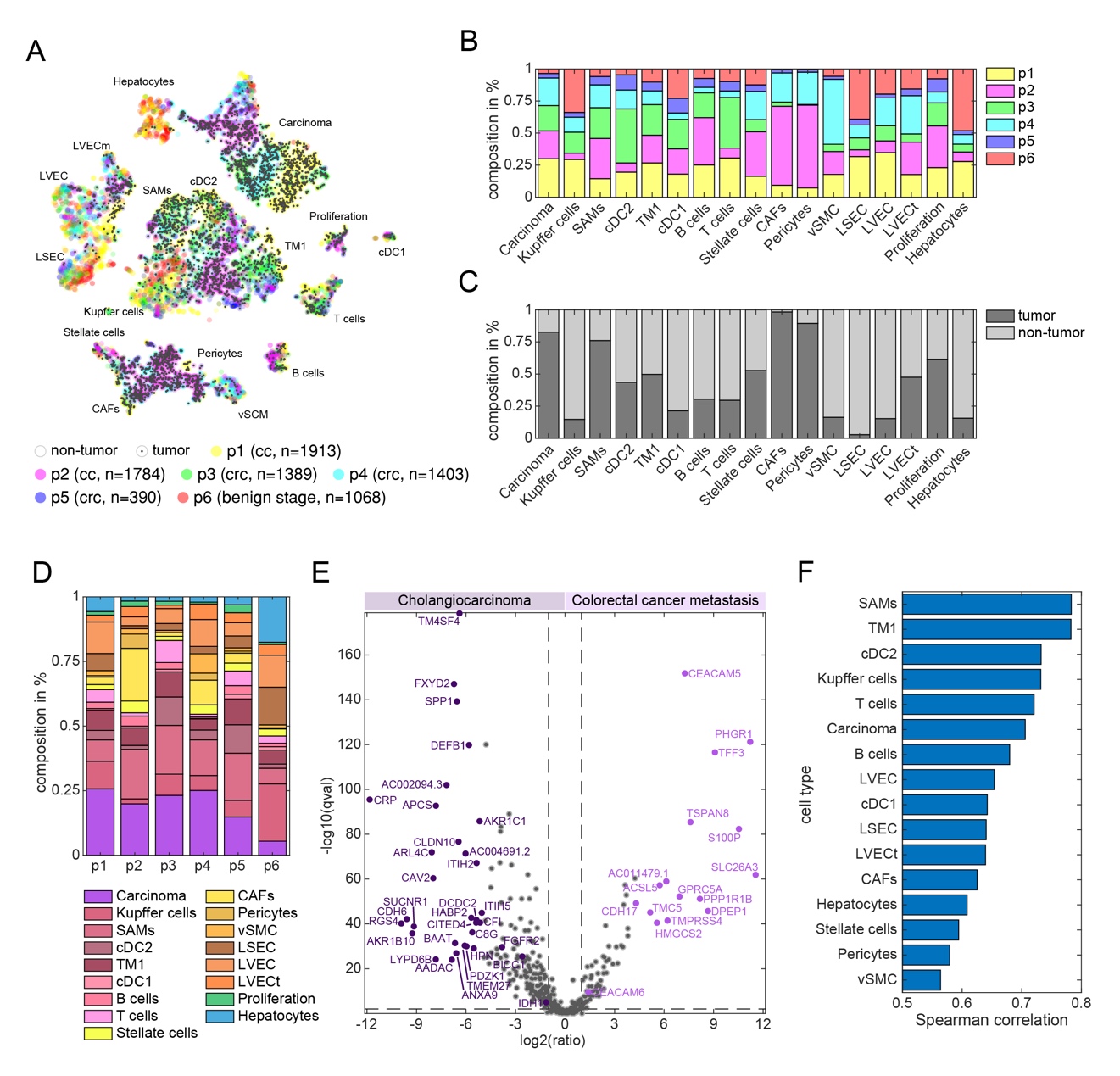


## Appendix Figure S1 – Patient distribution across all clusters

**(A)** tSNE plot of all Seurat clusters color coded by the patient of origin as detailed in Datasets EV1. ‘n’ - indicates the number of cells per patient **(B)** Relative composition of each cluster by patients. **(C)** Relative composition of each cluster by sample origin (tumor or non-tumor). **(D)** Distribution of cell types by patient. **(E)** Differential gene expression between the carcinoma cells from colorectal cancer metastases and cholangiocarcinoma patients. **(F)** Sorted spearman correlations of the mean gene expression of the proliferation cluster and the remaining 16 clusters demonstrate that it consists predominantly of immune cells.


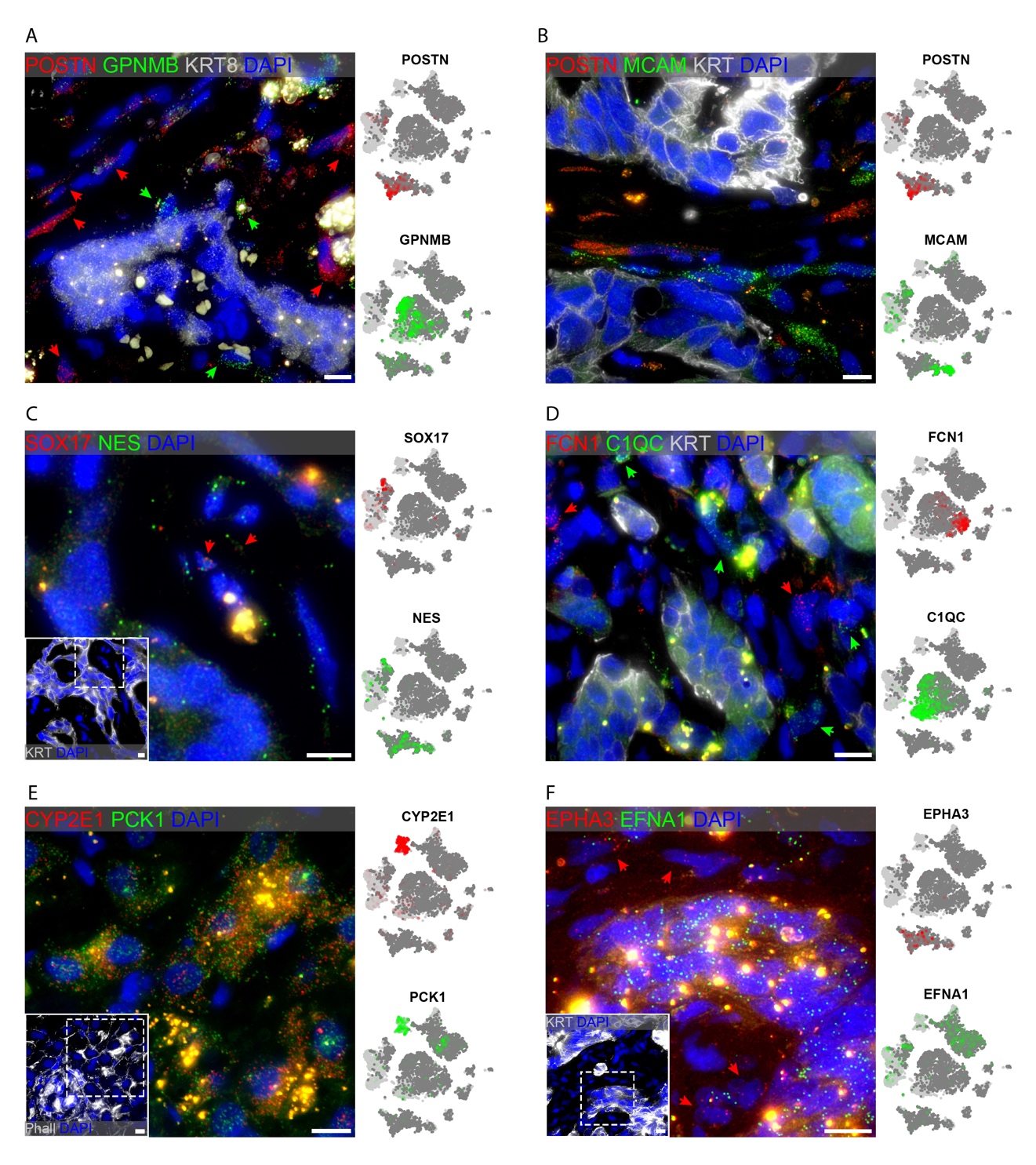


## Appendix Figure S2 – smFISH validations of the expression of key cell-type specific markers

**(A-B)** CAF marker POSTN, SAM marker GPNMB and vSMC marker MCAM, **(C)** Endothelial marker SOX17 and CAFs marker NES, **(D)** TM1 marker FCN1 and C1QC expressed in SAMs, **(E)** Hepatocytes markers CYP2E1, PCK1, **(F)** CAF marker EPHA3 and carcinoma-expressed EFNA1. KRT – pan-cytokeratin antibody staining marking the carcinoma cells. ‘Phall’ in panel E is Phalloidin membrane staining. Scale bar in all panels is 10um.


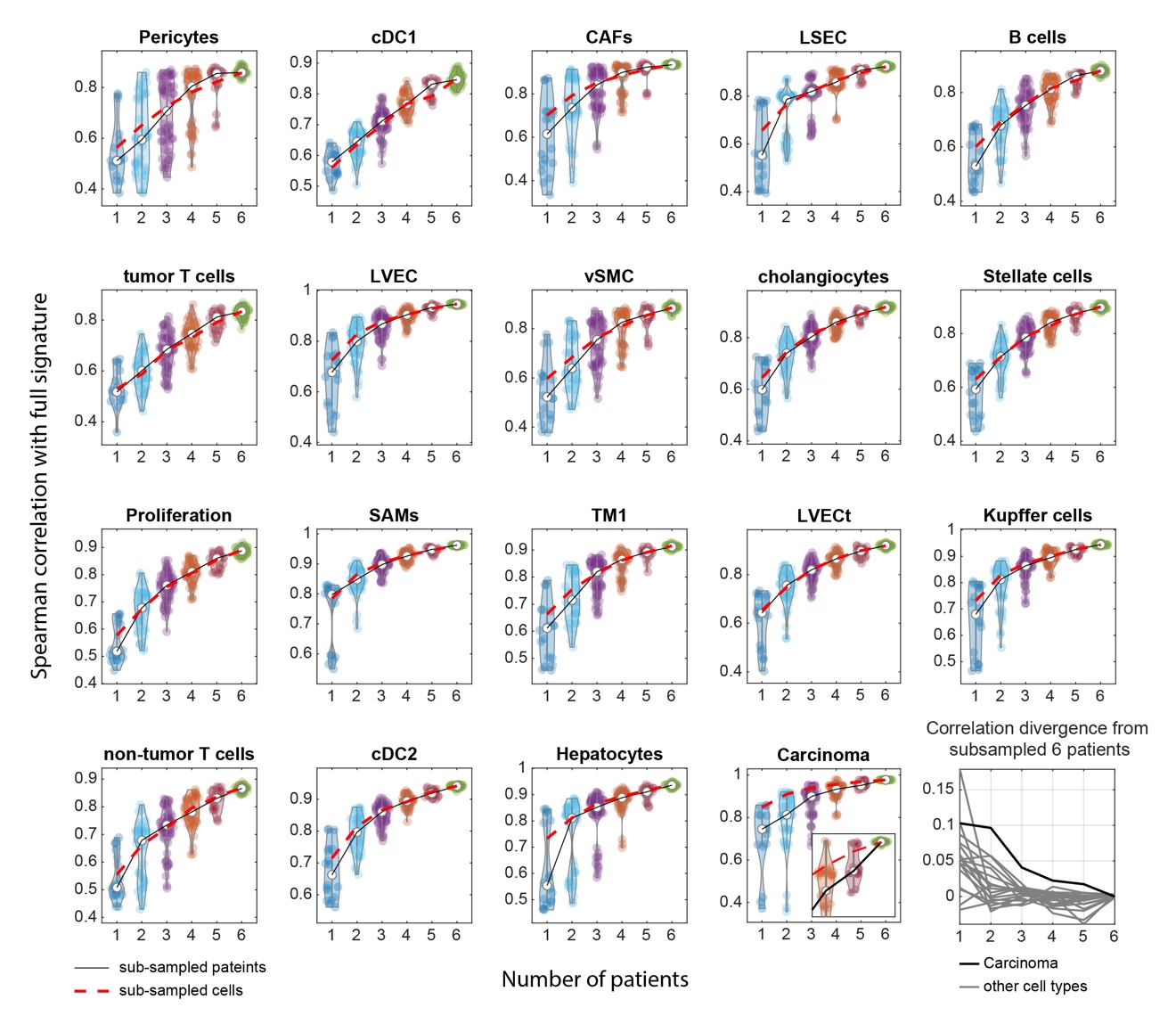


## Appendix Figure S3 – Stability analysis of the cluster gene expression signatures.

Spearman correlations between the mean gene expression signatures for each of the 19 cell types from sub-samples of the six patients (all possible combinations) to the mean gene expression of the full atlas (all six patients, solid black lines) or to an equally-sized sub-samples of cells from all six patients (dashed red line). Expression signatures converge for most cell types, beyond 3 patients, at 5-6 patients. Inset in carcinoma panel is a magnification of 4-6 patients sub-sampling, highlighting the divergence of the red dashed line the black solid line. The last panel shows the difference between the red dashed line and black line for carcinoma cells (black line) and the remaining TME cell types (gray lines).


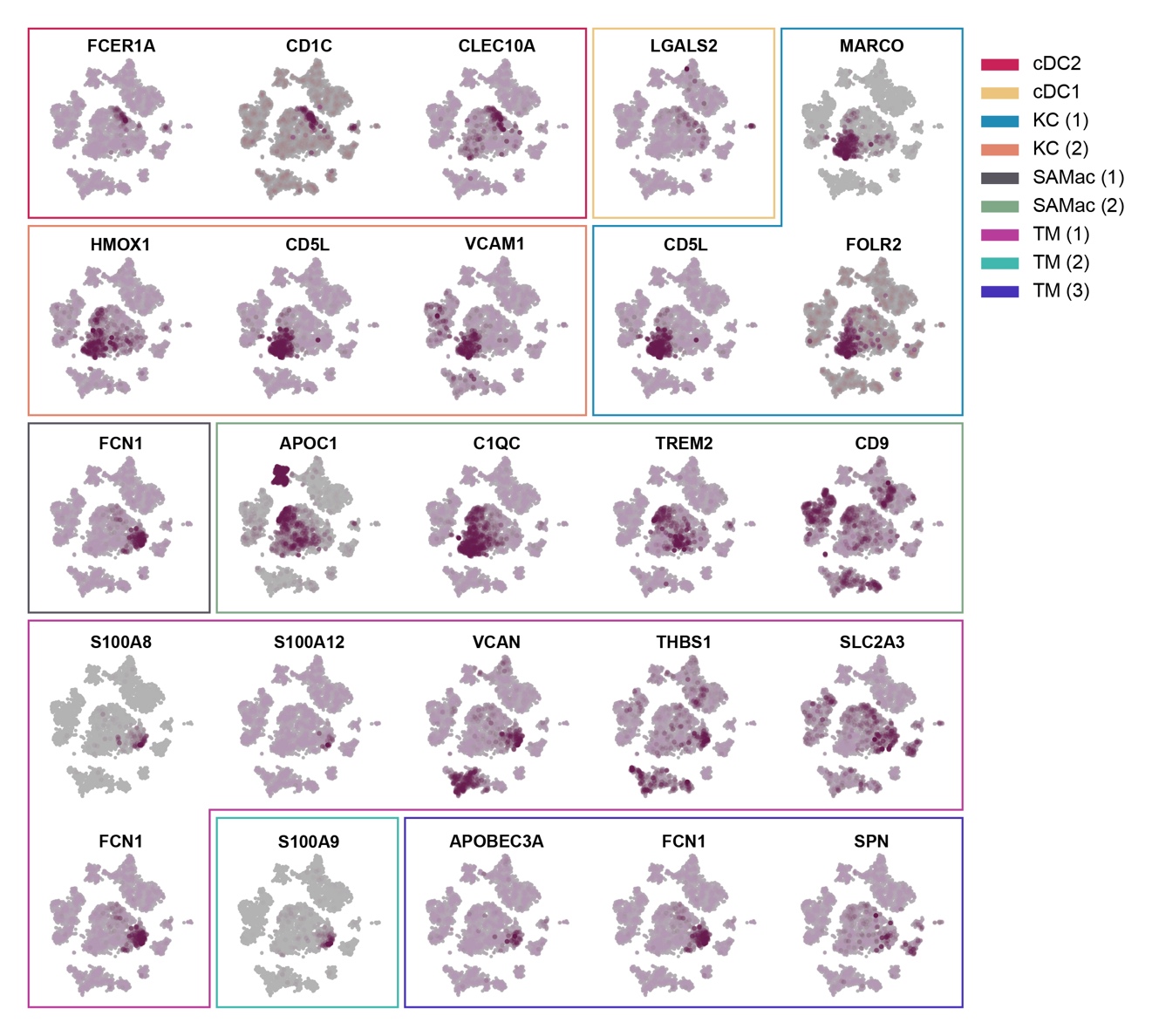


## Appendix Figure S4 – Immune cell type annotation

tSNE plots of genes used as cell type-specific markers in the cirrhotic liver study by Ramachandran *et .al.* Box colors denote the annotation of cell types from Ramachandran et al. (Ramachandran *et al*, 2019).


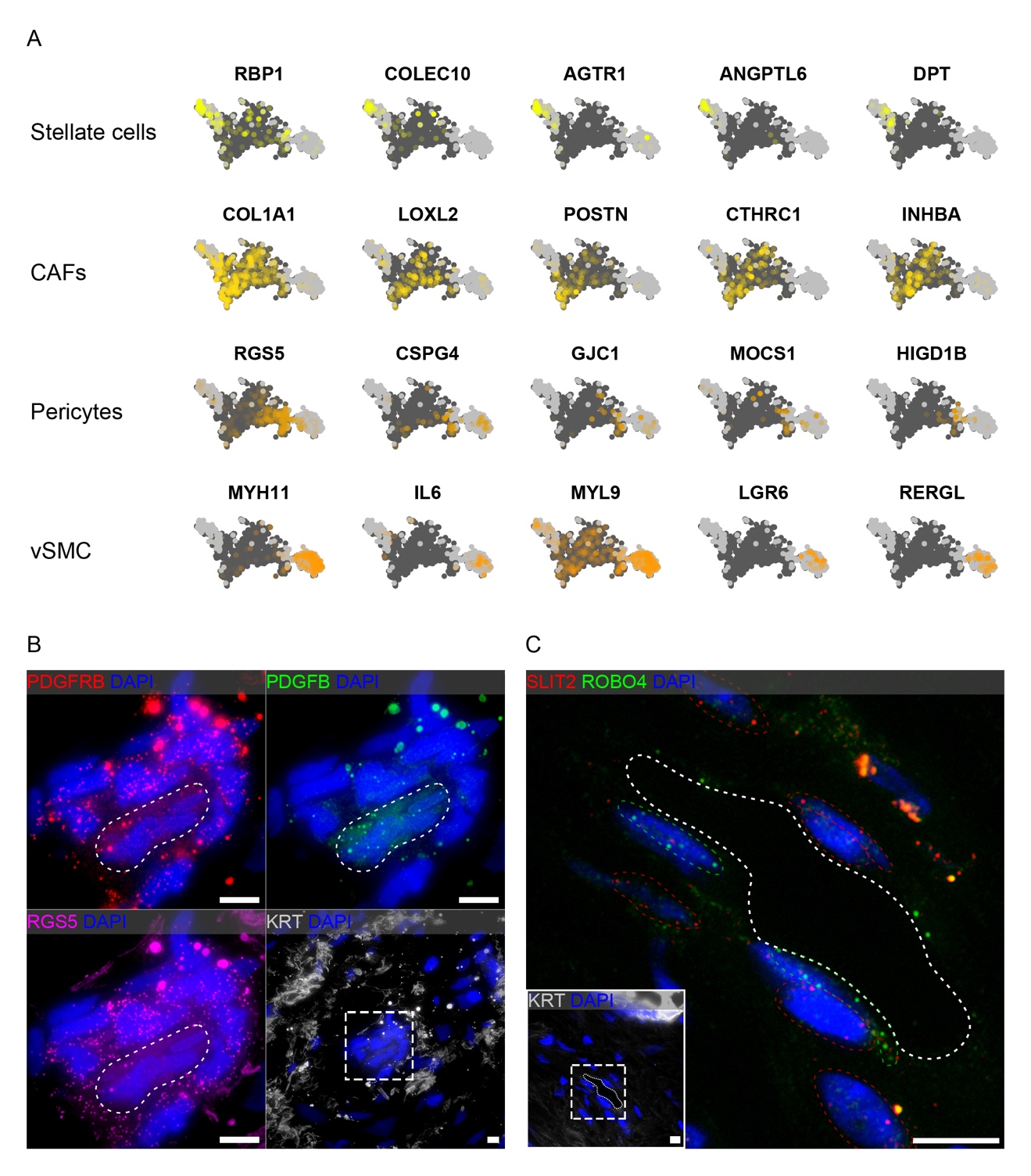


## Appendix Figure S5 – Markers of the mesenchymal cell types

**(A)** Markers for the four mesenchymal cell types, obtained by comparisons between each Seurat cluster and the remaining three clusters in the mesenchymal coarse-grained cluster. Rows denote the mesenchymal cell type (Stellate cells, CAFs, Pericytes, vSMC). **(B-C)** smFISH validation of genes mediating the interaction between pericytes and endothelial cells. **(B)** Tumor pericytes co-expressing RGS5 (magenta dots) and the ligand PDGFRB (red dots), and the endothelial-specific ligand PDGFB (green dots). Dashed line mark endothelial cells that are negative for RGS5. **(C)** Expression of the ligand SLIT2 mRNAs (red dots) in pericytes (marked by red dashed lines) and ROBO4 mRNAs (green dots) in endothelial cells (marked by green dashed lines). Blood vessel marked with dashed white line. Dashed box marking the blowup area. Scale bar in all panels is 10um. KRT – pan-keratin immunofluorescence signal highlights the carcinoma cells.


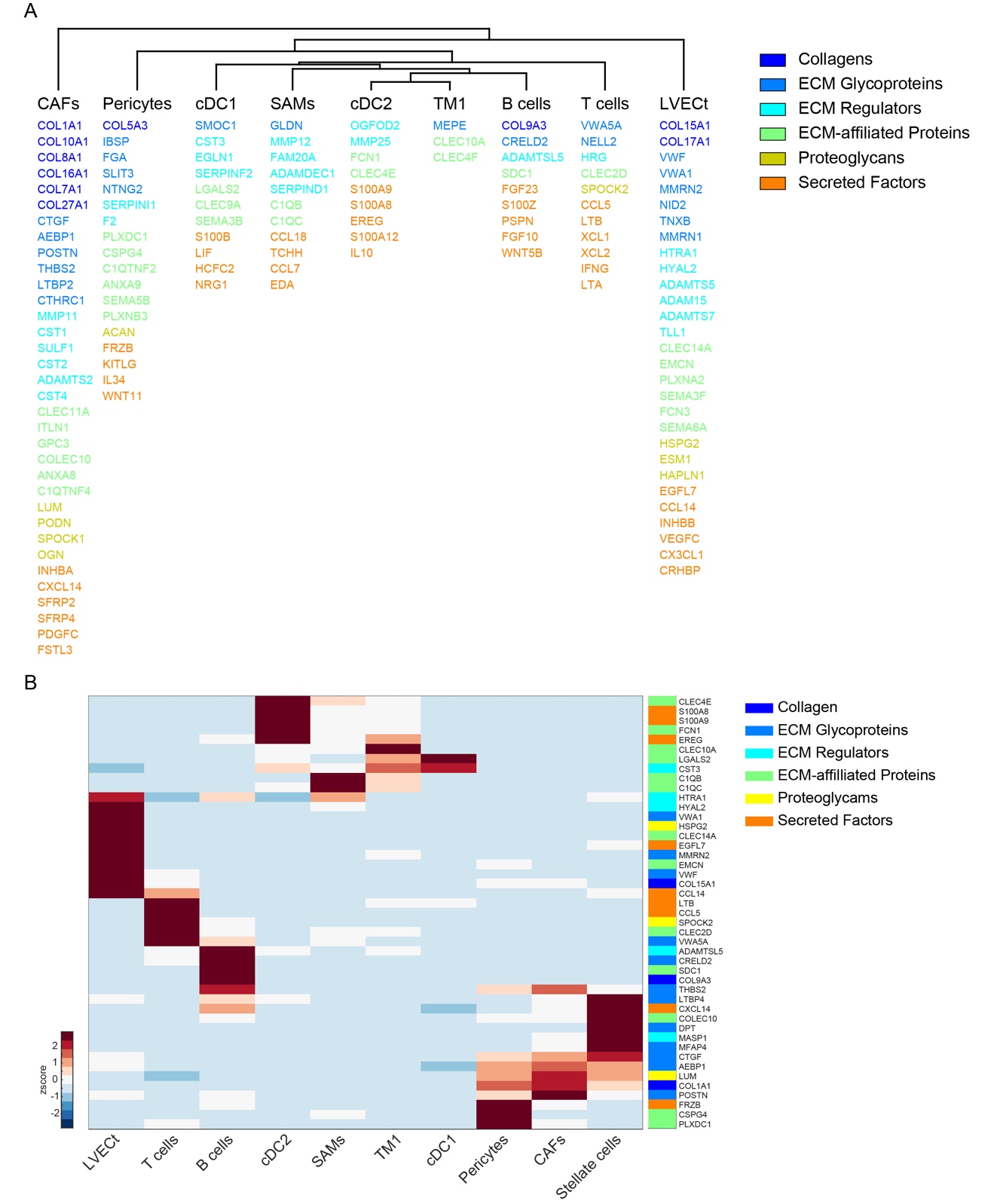


## Appendix Figure S6 – Matrisome analysis

**(A)** Dendrogram of matrisome genes specifically expressed in cancer cells of the TME. Matrisome subclasses are color coded. Genes from the same matrisome subclass are sorted by the mean expression of normalized cellular UMIs for each cell type. Up to six genes are shown per class. **(B)** Zscore value of the matrisome in tumor cell types found in the TME. Gene subclasses are color-coded. Stellated cells cluster was added as an out-group. Shown are genes with Zscore>1 or Zscore<-1.


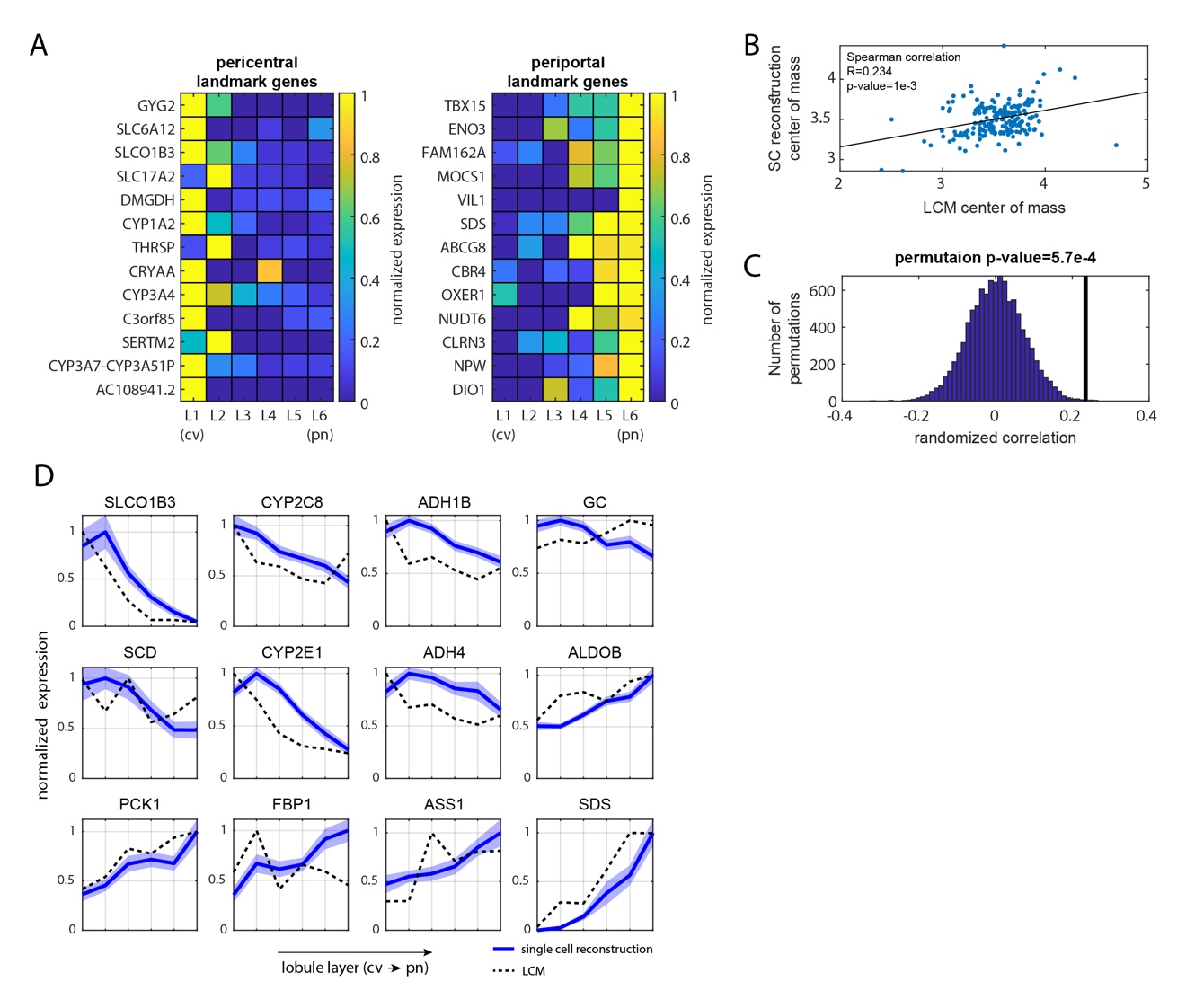


## Appendix Figure S7 – Hepatocytes spatial reconstruction

**(A)** Relative expression of the pericentral/periportal landmarks used for hepatocytes spatial reconstruction along the six lobule layers (labeled as L1 to L6 on the x-axis), spanning the central vein (cv) to portal node (pn). Expression values are normalized to the maximum expression across the layers. **(B)** Spearman correlation between the LCM center of mass and single cell (SC) center of mass. **(C)** Histogram of the randomized center of mass of the spatially reconstructed hepatocytes and center of mass of LCM for significantly zonated genes (see Methods). Black line is the correlation of the non-randomized set. P-value computed numerically as the fraction of permutations yielding a higher correlation. **(D)** Zonation profiles of selected genes shown as the mean expression across the six layers of the hepatic lobule. Solid blue line - single cells reconstructed zonation profiles, blue patches are standard errors of the mean. Dashed black line – zonation profiles from the LCM data.


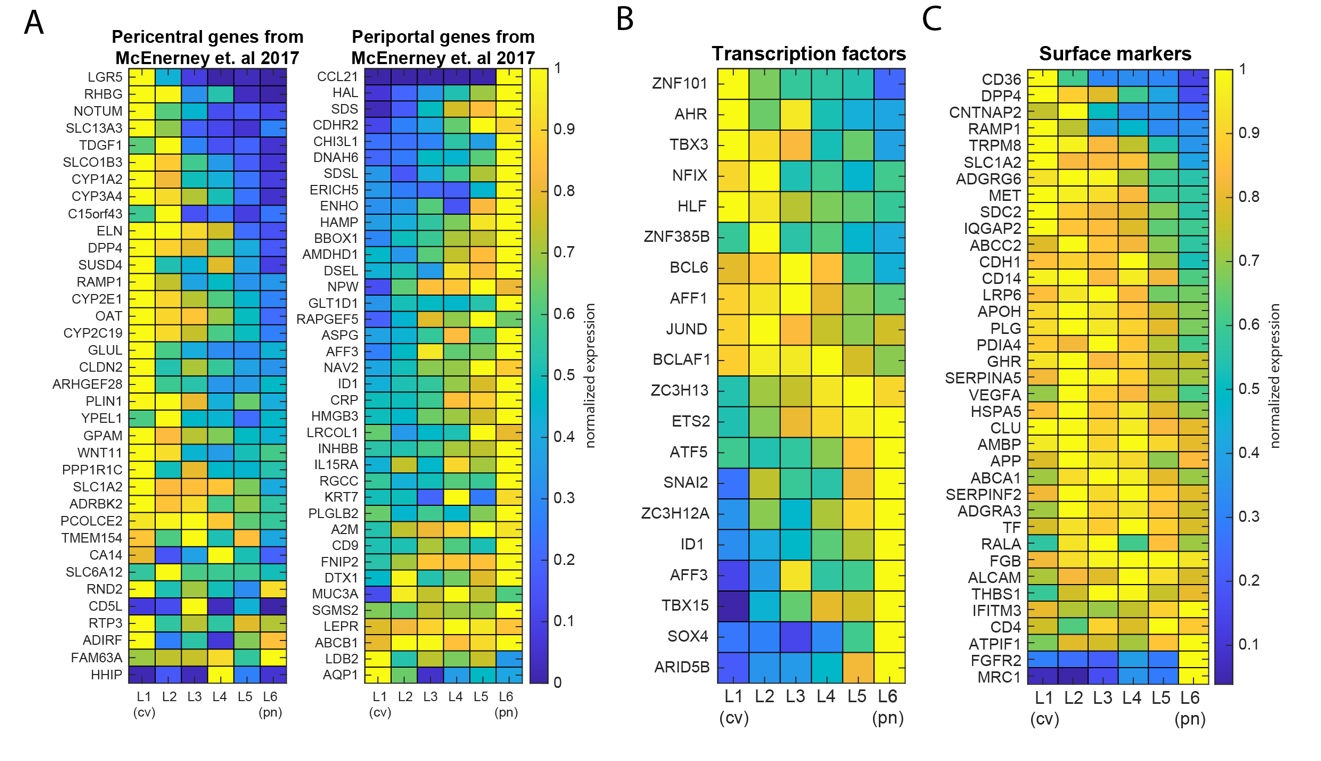


## Appendix Figure S8 – Spatial reconstruction validation, transcription factors and surface markers

**(A)** Validation of hepatocytes reconstruction using pericentral and periportal gene sets identified by McEnerney et al. (McEnerney *et al*, 2017). **(B-C)** Zonation of transcription factors **(B)** and surface markers **(C)** expressed in hepatocytes. Reconstructed layers across the central vein (cv) and portal node (pn) are marked by L1 to L6. (B-C) Shared color bar on the right. Expression values are normalized to the maximum expression a cross the layers.


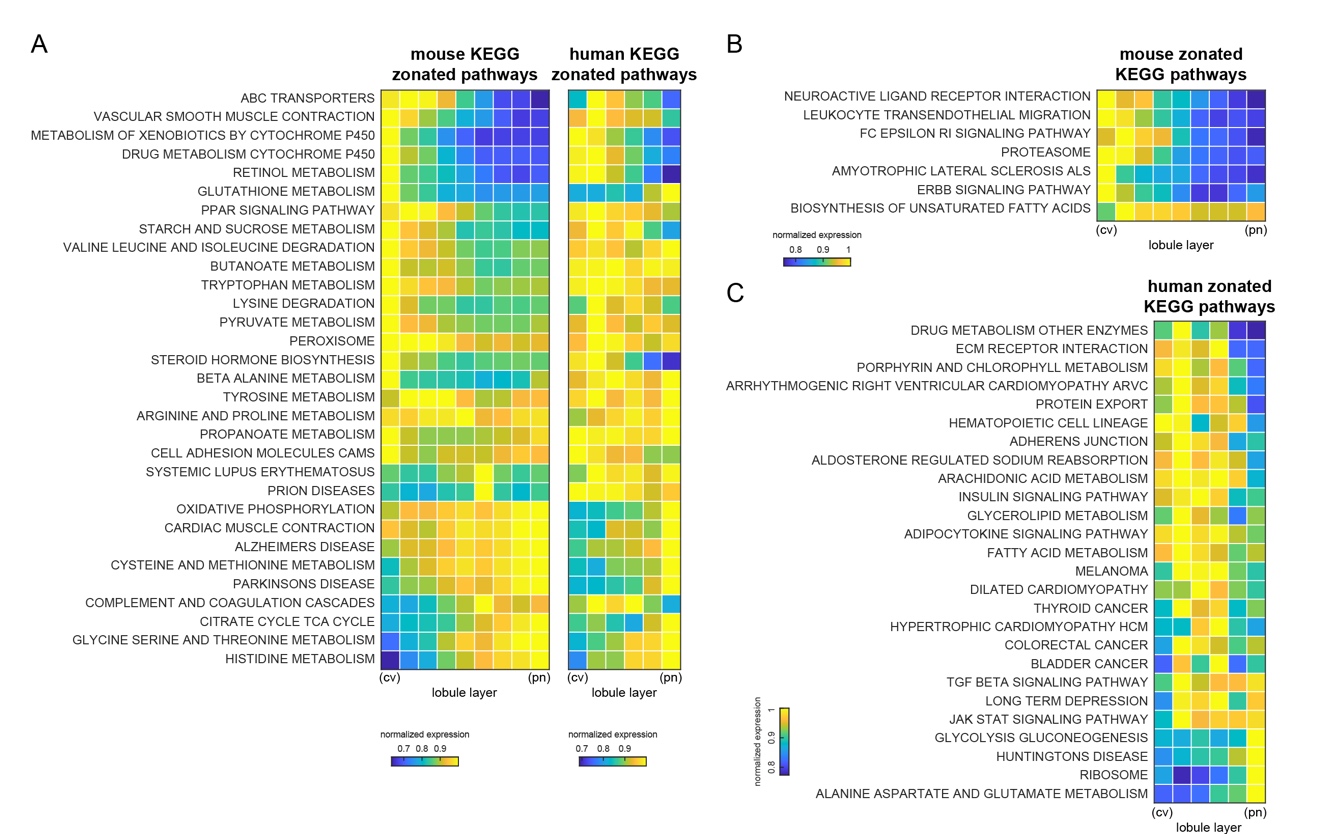


## Appendix Figure S9 – Comparison of Kegg pathways enrichment analysis between human and mouse

**(A)** Kegg pathway enriched in zonated genes in both human and mouse. Pathways are sorted by the centers of mass of the average zonation profile in mouse (Halpern *et al*, 2017). **(B-C)** Kegg pathway enriched in zonated genes in mouse but not in human **(B)** and human but not mouse **(C)**. Expression values are normalized to the maximum expression a cross the layers. Pathways profile are sorted by the center of mass from the central vein (cv) to the portal node (pn).

# References

Halpern KB, Shenhav R, Matcovitch-Natan O, Tóth B, Lemze D, Golan M, Massasa EE, Baydatch S, Landen S, Moor AE, *et al* (2017) Single-cell spatial reconstruction reveals global division of labour in the mammalian liver. *Nature* 542: 352–356

McEnerney L, Duncan K, Bang B-R, Elmasry S, Li M, Miki T, Ramakrishnan SK, Shah YM & Saito T (2017) Dual modulation of human hepatic zonation via canonical and non-canonical Wnt pathways. *Exp Mol Med* 49: e413–e413

Ramachandran P, Dobie R, Wilson-Kanamori JR, Dora EF, Henderson BEP, Luu NT, Portman JR, Matchett KP, Brice M, Marwick JA, *et al* (2019) Resolving the fibrotic niche of human liver cirrhosis at single-cell level. *Nature* 575: 512–518
